# Past present and future of radiosensitization in cervical cancer

**DOI:** 10.3389/fonc.2026.1846581

**Published:** 2026-06-19

**Authors:** Olivia N. Julian, Isabella Velarde, Sunil J. Advani, Jyoti Mayadev, Ramez N. Eskander

**Affiliations:** 1 Division of Gynecologic Oncology, Department of Obstetrics, Gynecology, and Reproductive Sciences, University of California, San Diego, Rebecca and John Moores Cancer Center, La Jolla, CA, United States; 2 University of California San Diego School of Medicine, La Jolla, CA, United States; 3 Department of Radiation Medicine and Applied Sciences, University of California San Diego, La Jolla, CA, United States

**Keywords:** antibody drug conjugate (ADC), immunotherapy, locally advanced cervical cancer (LACC), multimodal therapy, novel treatment approaches, radiation therapy

## Abstract

Cervical cancer remains a major health concern with over 662,000 cases and 348,000 deaths worldwide yearly. Treatment of early stage disease is generally surgical resection with excellent 5-year survival rates. Locally advanced cervical cancer (LACC) is potentially curable but still has only an approximately 50% 5-year survival rate. LACC is currently treated with chemoradiotherapy (cisplatin). Cisplatin as a radiosensitizer was established by 4 seminal, randomized clinical trials in the 1990s. While radiation techniques have advanced significantly over the past 25 years, the use of cisplatin as a radiosensitizer has not changed. With the development of new, more precise chemotherapy regimens in the form of antibody drug conjugates (ADCs), these drugs would have the ability to improve the therapeutic index of radiotherapy. While ADCs have proven improvement in overall survival and progression free survival in metastatic cervical cancer, they are yet to be studied in the LACC population.] Immune checkpoint inhibitors (ICIs) have also shown improved outcomes in use with metastatic cervical cancer and LACC. We feel that ICIs should be considered as an adjuvant therapy to the more precise ADC radiotherapy to improve tumor control. In summary, multimodal ADC-radio-immunotherapy may benefit patients with LACC and warrants investigation.

## Introduction

Cervical cancer is the fourth most common female cancer globally after breast, colorectal and lung cancer and remains a leading cause of cancer death among women (1, 2). Human papilloma virus (HPV) infection drives cervical malignant transformation with HPV 16 and 18 being the most common carcinogenic subtypes. Other oncogenic high risk HPV subtypes include 31, 33, 34, 35, 39, 45, 51, 52, 56, 58, 59, 66 and 68 (3). In 2022, there were 662,301 new cases of cervical cancer and 348,846 deaths and was ranked among the top three causes of cancer related deaths in 97 countries (4). If current trends persist, it is estimated that 760,082 new cases and 411,035 deaths will be attributable to cervical cancer globally by 2030 (4).

At diagnosis, approximately 50% of cervical cancer patients present with organ-confined early stage disease, about 5% present with metastatic disease and about 40% have more locally advanced disease (5, 6). Early stage cervical cancer includes FIGO stage IA, 1B disease and certain cases of stage IIA where tumor is confined to the cervix or upper vagina and is amenable to surgical resection. Current treatment for these patients is associated with high tumor control and favorable prognosis with >90% 5 year survival (7). At the other extreme, FIGO stage IVB metastatic cervical cancer that has spread to distant organs is treated with systemic chemotherapies and patient survival drops to <20% (7). In between these two extremes, a significant number of women present with non-metastatic locally advanced cervical cancer that includes FIGO stage IIA2 to IVA (6, 8). While having disease locally spread beyond the cervix, these cancer patients are potentially curable, however their 5 year survival rate is only approximately 60% with current standard of care of concurrent chemo-radiotherapy (7).

Radiotherapy is utilized as a component of curative intent therapy for LACC. Mechanistically, ionizing radiation (IR) delivered by radiotherapy induces DNA damage including lethal DNA double strand breaks that results in cell kill. The therapeutic index of radiotherapy is governed by differential DNA damage response and repair processes that preferentially kill cancer cells as opposed to damaging normal tissue. Early radiobiological studies established the therapeutic value of delivering IR as multiple smaller daily fractions compared to a single larger fraction (9, 10). Tumor control by fractionated radiotherapy is influenced by DNA damage repair responses, tumor repopulation by proliferation of surviving tumor cells, cancer cell redistribution through the cell cycle and reoxygenation of hypoxic tumor regions. These concepts of repair, redistribution, reoxygenation and repopulation are called the “4 R’s” of radiotherapy (11). Repair and repopulation are sources of resistance to radiotherapy that result in treatment failure where DNA strand breaks are repaired or cells that were not initially killed begin to proliferate leading to an increase in radioresistant tumor cells (11). In contrast, redistribution and reoxygenation are two mechanisms that sensitize cancer cells to IR kill where cells are radiated on different days to maximize cells that are in the G_2_/M phase and allowing necrotic areas to be re-vascularized, both of which increase cellular response to radiation (11). More recently, additional “Rs” have been proposed including radiosensitivity, rate of dose and reactivation of anti-tumor response (12). While the original “4 Rs” are well established and form a guiding principle of delivering radiotherapy over the course of multiple daily fractions, tumor heterogeneity is a major cause of treatment resistance (both intrinsic and acquired) and remains a formidable clinical problem that results in treatment failure and poor patient outcomes (13).One approach to increase the therapeutic window of radiotherapy is by using radiosensitizing drugs that increase irradiated cell kill. Mechanistic understanding of IR induced DNA damage and cellular repair processes has identified pathways that can be pharmacologically inhibited that include DNA-PK, PARP and ATM (14). An advantage of DNA damage response and repair inhibitors over chemotherapies is that they lack inherent cytotoxicity and are only active in irradiated cells experiencing DNA damage. Unfortunately, clinical trials reported to date evaluating DNA damage response inhibitors in combination with radiotherapy have not proven effective at improving tumor control. Of particular concern, these trials revealed significant clinical shortcomings of DNA damage response inhibitors in that they caused increased normal tissue damage of sensitive critical structures adjacent to the irradiated tumor target (15–18). This in part may be explained by the fact that the lack of tumor-targeted radiosensitization results in radiosensitization of not only cancer cells but also peri-tumoral critical normal structures which negates improving the therapeutic index with radiotherapy. Here, we discuss the rationales for combining radiotherapy with tumor-targeted cytotoxins and immunotherapies for women with locally advanced cervical cancer. Such a multi-modal precision oncology strategy can harness therapeutic synergies to improve cervical cancer patient outcomes.

## Concurrent chemo-radiotherapy

**Figure 1:**
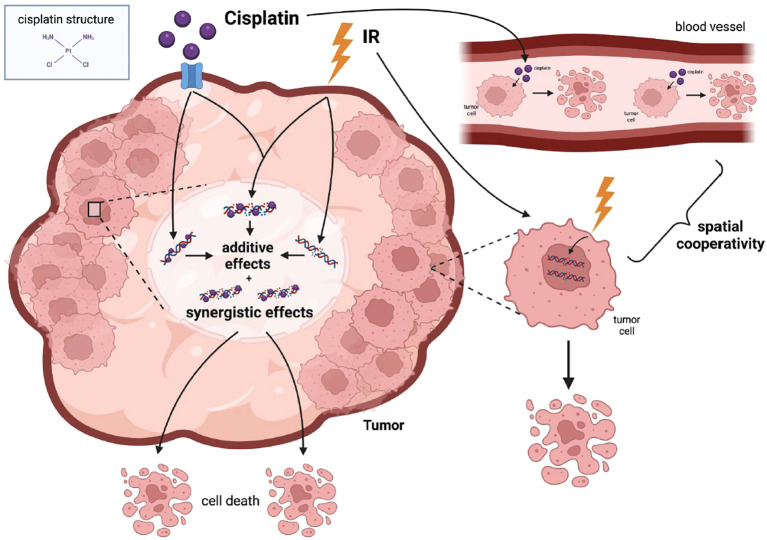
Rational for concurrent chemoradiotherapy. Cisplatin will cause DNA damage via DNA crosslinking while radiation will cause DNA damage by single or double strand breaks. Using two mechanisms of DNA damage will cause both an additive effect and a synergistic effect where each mode of therapy will increase the DNA damage from the other and greater than the sum or their individual effects if used alone. Additionally, chemoradiation applies spatial cooperativity where radiation will target local cancer cells while chemotherapy will target micrometastases.

As cervical cancers progress locally and spread to regional pelvic lymph nodes, extended radiotherapy treatment fields are needed to fully encompass larger target volumes that result in significant irradiation to greater portions of sensitive normal tissues that include the bladder, small intestines, rectum and vagina. However, radiotherapy dose is constrained by damage to the surrounding organs at risk (19). One strategy to overcome this problem and improve patient outcomes for locally advanced cancers is to combine complementary treatments that can synergize. A highly successful example of such a strategy is the concurrent chemo-radiotherapy paradigm where systemic chemotherapies are given together with focal radiotherapy. Delivering chemotherapy in combination with radiotherapy has multiple strengths. First, cytotoxic chemotherapies and radiotherapy kill cells by different mechanisms. Having non-overlapping mechanisms of cancer cell kill can overcome therapy resistance due to inherent cancer cell heterogeneity within tumors (20) (**Figure 1**). In addition, systemically delivered chemotherapies and focal radiotherapy provide spatial cooperativity. Radiotherapy is directed at visualized macroscopic tumors and chemotherapy attacking microscopic cancer spread out of the irradiated field (20). Finally, certain drugs are radiosensitizers that synergize with irradiation to increase IR induced DNA damage (21).

The current standard of care of concurrent chemo-radiotherapy in locally advanced cervical cancers was established by four landmark randomized Phase III clinical trials from the 1990s (Radiation Therapy Oncology Group (RTOG) 9001, Gynecologic Oncology Group (GOG) 120, Southwest Oncology Group (SWOG) 8797 and GOG 123) (22–25). These practice changing studies unequivocally demonstrated the superiority of combining chemotherapy with radiotherapy. Importantly, all four trials showed improved overall survival (OS), progression free survival (PFS) and disease free survival (DFS) benefit in cervical cancer patients when chemotherapy was combined with radiotherapy and have dictated patient care over the last 20 years (**Table 1**).

**Table 1:** Randomized clinical trials for concurrent chemoradiotherapy.

Study name	Included patients	Study arms	Findings
RTOG 9001	Stage IIB-IVA (n = 403)	**Arm A:** Pelvic and para-aortic RT **Arm B:** Pelvic RT + concurrent cisplatin + 5 FU	**Chemoradiation improved PFS and OS** compared to RT alone (22)
GOG 120	Stage IIB-IVA (n=526)	**Control**: EBRT + hydroxyurea **Arm A:** EBRT + cisplatin + 5FU + hydroxyurea **Arm B:** EBRT+ cisplatin	**Cisplatin chemoradiation arms increased PFS and OS.** Cisplatin alone arm with fewer side effects than multi drug chemotherapy regimen (23)
GOG 123	Stage IB2 (n=396)	**Arm A:** RT alone → hysterectomy (TAH) **Arm B:** RT + cisplatin → TAH	**Cisplatin+ RT followed by TAH significantly reduced disease recurrence and OS** compared to RT alone (24)
SWOG 8797	Stage IA2, IB, IIA after radical hysterectomy (n = 268)	**Arm A**: adjuvant pelvic RT **Arm B:** adjuvant pelvic RT + concurrent cisplatin + 5 FU	**Chemoradiation improved PFS and OS** compared to RT alone (25)

Bold values highlight the arms of the study and the overall summative findings of the trial.

RTOG 9001 and GOG 120 both specifically focused on the locally advanced cervical cancer patient population, i.e. stage IIB-IVA. RTOG 9001 tested extended field radiation therapy alone that included pelvic and para-aortic nodes versus a more limited pelvic radiotherapy target volume combined with cisplatin and 5-fluorouracil (5-FU). This study demonstrated improved patient overall survival and decreased tumor recurrence in the chemo-radiotherapy arm compared to radiotherapy alone. Moreover RTOG 9001 showed the advantage of spatial cooperativity. Systemic chemotherapy with a smaller and more focused pelvic radiotherapy field produced superior outcomes and reduced radiation to normal tissues that are the consequence of an extended para-aortic radiotherapy field (22). Given the hematological and gastrointestinal toxicities of administering hydroxyurea (HU) with radiotherapy, GOG 120 tested if cisplatin containing regimens given together with radiotherapy could improve patient outcomes. This study randomized irradiated cervical cancer patients to three different chemotherapy regimens including HU alone, cisplatin alone or a more intensive cisplatin + 5 FU + HU triplet. Both cisplatin containing arms showed superior OS and PFS in cervical cancer patients compared to HU alone. Importantly in combination with radiotherapy, cisplatin alone was just as effective as the triple drug regimen of cisplatin + HU + 5-FU with the advantage of significantly less toxicity (23). GOG 120 published in 1999 was practice defining and resulted in the current standard of care for locally advanced CC. The superiority of concurrent chemo-radiotherapy was further extended to cervical cancer patients with adverse pathologic features such as bulky stage IB tumors >4 cm or patients status post radical hysterectomy with positive pelvic lymph nodes, positive margins or microscopic parametrial involvement. Historically, such patients were treated with either neoadjuvant or adjuvant radiotherapy before or after surgical resection. Both SWOG 8797 and GOG 123 tested if adding chemotherapy to radiotherapy would be beneficial to this high risk patient population. In both studies, concurrent chemo-radiotherapy resulted in improved outcomes compared to radiotherapy alone (24, 25) (**Table 1**). These seminal clinical trials demonstrated that comprehensively tackling the known macroscopic tumor, regional lymph node spread and potential metastatic seeding with multimodal cooperative tumor killing by chemotherapy and radiotherapy improved outcomes for cervical cancer patients.

Of note, these paradigm shifting trials from the 1990s combined non-targeted cytotoxic chemotherapies with crude 2-dimensional and 3-dimensional external beam radiotherapy techniques reliant on bony anatomy (26). In the ensuing decades, radiation planning and delivery technologies have grown increasingly sophisticated to advance toward realizing precision oncology treatment (27). Intensity modulated radiotherapy (IMRT) conforms and shapes IR deposition to tumors that are more clearly delineated by positron emission tomography-computed tomography (PET-CT) and magnetic resonance imaging (MRI) (28). Image guided radiotherapy (IGRT) corrects for daily set up variations in patient anatomy to ensure that each daily radiotherapy fraction over the course of multiple weeks is delivered to the planned target volume. In addition to more accurately targeting IR to tumors, IMRT and IGRT technologies reduce normal tissue damage in the irradiated pelvis (29). Brachytherapy, a treatment that is inherently more precise due to placing radiation sources directly within the tumor, has also undergone technological advancements. Older 2-dimensional brachytherapy planning techniques now employ medical physics advancements to more accurately deliver IR dose to the tumor target and minimize dose to peri-tumoral normal tissues (30). While radiation techniques have become more precise, the chemotherapy given with radiotherapy remains unchanged with the use of cisplatin. Although cisplatin is clearly effective when given with radiotherapy, such non-targeted cytotoxins increase normal tissue damage in the irradiated field and have systemic toxicities. Therapy-induced side effects such as bone marrow suppression, kidney damage, hearing loss and peripheral neuropathy cause treatment delays and dose reductions that negatively impact tumor control and patient quality of life (31, 32). Additionally, non-targeted chemotherapies do not leverage tumor vulnerabilities that drive tumor pathogenesis. Here, we discuss the rationale for combining radiotherapy with antibody drug conjugates (ADCs) carrying anti-tubulin monomethyl auristatin E (MMAE) payloads with radiotherapy for women with locally advanced cervical cancer. In addition to having inherent potent cytotoxic activity, ADCs can restrict MMAE radiosensitization to cervical cancer cells while simultaneously sparing adjacent normal tissues. Such a precision oncology chemo-radiotherapy strategy can improve cervical cancer patient outcomes.

## Antibody drug conjugates

Antibody drug conjugates (ADCs) are a rapidly expanding class of targeted cancer therapy (33). ADCs are designed to deliver potent cytotoxins with spatial precision in a receptor restricted fashion while simultaneously minimizing normal tissue damage. ADCs are composed of monoclonal antibodies attached to potent cytotoxic drug payloads through chemical linkers (34). ADCs split the roles of tumor-specificity and killing into two distinct tasks. After intravenous injection, tumor-targeting is achieved by the antibody moiety recognizing membrane receptors that are overexpressed on cancer cells compared to normal tissues (35). Binding of ADCs to target membrane receptors triggers receptor-mediated endocytosis and intracellular trafficking through the endo-lysosomes. Within the acidic environment of lysosomes the chemical linker within ADCs is cleaved enzymatically or through pH dependent mechanisms. Cleavage of the ADC linker releases the cytotoxic drug payload that then kills cancer cells in a receptor-restricted manner.

In addition to targeted cell kill, ADC tumor control is amplified by the bystander effect where the released drug can diffuse into adjacent cancer cells in a receptor-independent manner (36). ADC drug payloads most commonly are anti-tubulins or topoisomerase I inhibitors (37). Anti-tubulins are either derived from auristatins (MMAE, MMAF) or maytansinoids (DM1, DM4). Topoisomerase I inhibitors are camptothecin based and include SN38 and a more potent derivative DXd. These anti-tubulins and topoisomerase I inhibitors share the property of being highly potent cytotoxins that are chemically amenable to antibody conjugation. In constructing ADCs, a fixed number of drug molecules are attached to each antibody, resulting in a characteristic drug-antibody ratio (DAR) (36). Based on optimization studies, anti-tubulin based ADCs typically have a DAR of 4. In contrast, topoisomerase I inhibitor based ADCs have up to 7–8 drug molecules attached to each antibody that results in twice as many drug molecules delivered compared to MMAE containing ADCs (38).

Initial clinical success of ADCs was limited to leukemias and lymphomas, but efficacy across diverse solid tumor histologies is being demonstrated, resulting in an expanding number of ADCs approved in cancers that are treated with concurrent chemo-radiotherapy. To date, there are at least 15 FDA approved ADCs (39) including the approval of tisotumab vedotin-ttfv (Tivdak) for cervical cancer. Tisotumab vedotin – ttfv is a tissue factor targeted ADC with the anti-tubulin monomethyl auristatin E (MMAE) as its drug payload. Since tissue factor is over-expressed in cervical cancer, Tivdak was tested in women with recurrent or metastatic cervical cancer in the phase III InnovaTV-301 trial. Excitingly, Tivdak achieved an improved OS as second or third line therapy compared to investigator’s choice chemotherapy in this difficult to treat patient population and gained FDA approval as the first ADC for cervical cancer (40).

While tissue factor targeted Tivdak is currently the only ADC approved for cervical cancer, other ADC receptor targets are also overexpressed on cervical cancer cells. In addition to tissue factor, cervical cancers have been shown to overexpress TROP-2, HER-2 and Nectin-4 suggesting that current FDA approved ADCs for other cancer histologies may be effective in cervical cancer (41–44) (**Table 2**). The TROP-2 receptor is targeted by toposiomerase I loaded ADCs sacituzumab govitecan (Trodelvy) and datopotamab deruxtecan (Datroway). The HER-2 receptor is targetable with an anti-tubulin ADCs trastuzumab emtansine (Kadcyla) and Disitamab vedotin (Aidixi) or the topoisomerase I inhibitor ADC trastuzumab deruxtecan (Enhertu). Finally, Nectin-4, like tissue factor, can be targeted by the MMAE containing ADC enfortumab vedotin (Padcev). Excitingly, two TROP-2 ADCs (sacituzumab tirumotecan and sacituzumab govitecan) are being evaluated in cervical cancer. Sacituzumab tirumotecan (sac-TMT) showed promising results in a phase II trial and now is undergoing a phase III trial as second line treatment for recurrent or metastatic cervical cancer compared to standard of care chemotherapy in the TroFuse-020 trial (45). In the phase 2 basket study EVER-132-003, sacituzumab govitecan showed an ORR of 43% in the subset of 40 recurrent or persistent cervical cancer patients (46). Given the excitement surrounding ADCs based precision oncology therapy and proof of concept efficacy of tisotumab vedotin, multiple clinical trials are underway to test the activity of ADCs targeting alternative receptor targets in cervical cancer and to deliver anti-tubulin or topoisomerase I inhibitor drug payloads (45, 47–50) (**Table 3**).

**Table 2:** FDA approved ADCs with potential in cervical cancer.

ADC name	Target receptor	Linker chemistry	Payload	Drug mechanism of action	DAR*
Tisotumab vedotin (Tivdak)	Tissue Factor	Cleavable- valine citrulline	MMAE*	Anti-tubulin	4
Sacatuzumab govitecan (Trodelvy)	TROP2	Hydrolyzable and cleavable	SN-38*	topoisomerase -1 inhibitor	7-8
Datopotumab deruxtecam (Datroway)	TROP2	Tetrapeptide cleavable	DXd*	topoisomerase -1 inhibitor	4
Disitamab vedotin (Aidixi)	HER2	Cleavable- valine citrulline	MMAE	Anti-tubulin	4
Trastuzumab deruxtecan (Enhertu)	HER2	Tetrapeptide Cleavable	DXd	topoisomerase -1 inhibitor	8
Trastuzumab emtansine (Kadcyla)	HER2	Non cleavable SMCC*	DM1	Anti-tubulin	3.5
Enfortumab vedotin (Padcev)	Nectin-4	Cleavable- valine citrulline	MMAE	Anti-tubulin	4
Mirvetuximab soravtansine	Folate Receptor Alpha	Cleavable- sulfo-SPDB*	DM4*	Anti-tubulin	3.5

*DAR: Drug antibody ratio, the number of conjugated drug molecules to each antibody.

*MMAE: Monomethyl aurastatin E.

*SN-38: 7-ethyl-10-hydroxycamptothecin.

*DXd: Deruxtecan.

*DM4: Ravtansine.

*SMCC: N-succinimidyl-4-(N-maleimidomethyl)cyclohexane-1-carboxylate.

*sulfo-SPDB: N-succinimidyl 4-(2-pyridyldithio)butane-2-sulfonic acid.

**Table 3:** ADCs in development for cervical cancer.

ADC name	Target antigen	Linker	Drug mechanism of action	DAR
Sacituzumab tirumotecan	TROP 2	Cleavable	Topo-1 inhibitor	7-8
ADRX-0706	Nectin-4	Cleavable	Anti-tubulin	8
Bulumtatug Fuvedotin (9MW2821)	Nectin-4	Cleavable	Anti-tubulin	4
LCB84	TROP2	Cleavable	Anti-tubulin	4
SBT6050	HER2	Cleavable	TLR8 agonist	Not available

Extending beyond cervical cancer, folate receptor alpha targeted ADC, mirvetuximab soravtansine, was FDA approved in ovarian, fallopian tube and peritoneal cancer after proof of benefit in the SORAYA and MIRASOL studies (51, 52). Interestingly, a retrospective trial of 123 cervical cancer patients showed that all of the primary tumors that were resected expressed folate receptor alpha (FR-a). High FR-a expression (>/= 2+ intensity) was found in 25% of these patients and there was significantly higher staining in metastatic tumors (53). These results suggest mirvetuximab soravtansine may prove efficacious in cervical cancer in addition to other gynecological cancers.

While ADCs are highly targeted drugs that systemically seek and kill cancer cells, the clinical experience has revealed dose limiting toxicities. ADC toxicities can be broadly categorized as on or off target. ADC targeted receptors are not exclusively expressed on cancer cells. This leads to payload drug delivery to normal tissue leading to “off target” toxicity. Additionally, “off-target” effects occur independent of target receptor engagement. Mechanisms include linker instability prematurely releasing drug, non-specific ADC internalization by attaching non-targeted receptors, bystander effects occurring when ADC cleavage in target cells releases the drug from antibody constraint which can diffuse into adjacent normal tissues (54, 55). In a recent meta-analysis, the incidence of significant ADC toxicities (grade ≥ 3) was reported to be >40% and included hematologic and neuropathic toxicity (54, 56).

Despite these drawbacks, ADCs have proven efficacious in multiple randomized clinical trials. To date, clinical trial testing of ADCs in cervical cancer have focused on recurrent or metastatic cervical cancer. This leaves an opportunity to investigate ADCs in the upfront curative locally advanced setting where ADCs in combination with radiotherapy could offer a precision oncology based chemo-radiotherapy strategy. Of the FDA approved ADCs, **Table 2** lists the ADCs that target receptors known to be overexpressed in cervical cancer.

## Immunotherapy

Cancer is characterized by genomic instability in which mutations and structural alterations result in tumor progression and metastases (57). These variations within cancer cells would be expected to give rise to tumor associated antigens that the immune system could recognize as “non-self” and mount an immune response to eliminate these cells (58). Curiously, tumors are able to evade recognition by the immune system in part through creating an immuno-suppressive environment. For example, immune checkpoints are proteins on the surface of immune cells that regulate the immune response and prevent immune attack of healthy tissues. Many tumors are able to activate immune checkpoints that block anti-tumor adaptive immune responses mediated by CD8+ T-cells (59).

**Figure 2:**
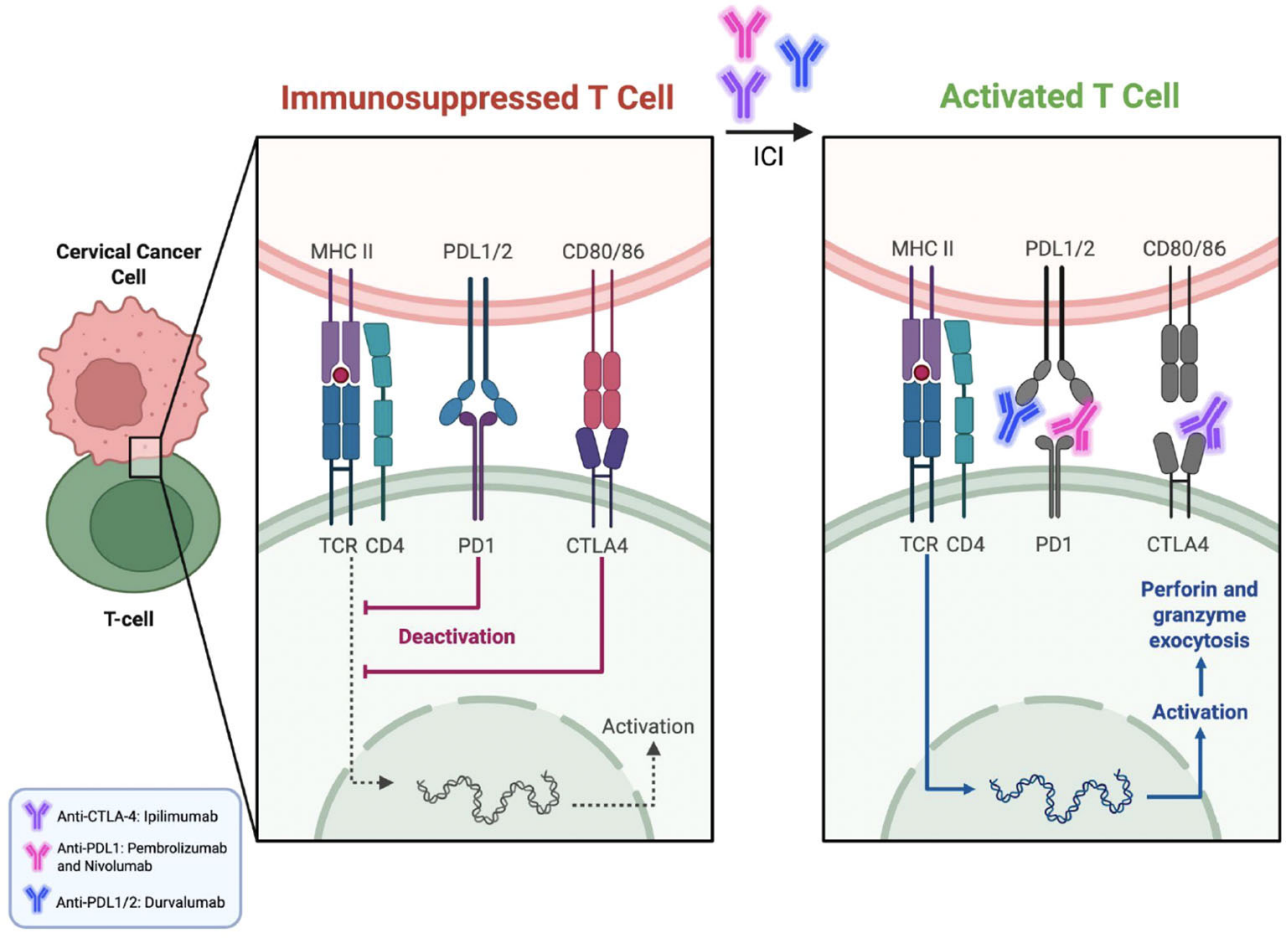
Immune checkpoint inhibitor mechanism. Cancer cells will learn to express ligands that lead to an immunosuppressed environment. PDL1/2 on the cancer cell will bind PD1 on the T cell or CD80/86 on the cancer cell will bind CTLA4 which both lead to a deactivation of the T cell. Immune checkpoint inhibitors block the ligands that lead to immunosuppression from binding, therefore leading to an appropriate host immune response to the cancer cells. *ICI: immune checkpoint inhibitor, *PD1: programmed cell death protein 1, *CTLA4: Cytotoxic T-Lymphocyte-Associated Protein 4, *PDL1/2: programmed death ligand 1/2, *TCR: T cell receptor, *MHC II: Major Histocompatibility Complex Class II, *CD4: Cluster of Differentiation 4, *CD80/86: Cluster of Differentiation 80/86.

The two most well characterized immune checkpoint pathways modulated in tumors and targetable in cervical cancer patients are cytotoxic T lymphocyte antigen-4 (CTLA-4) and programmed death-1 (PD-1) (**Figure 2**). The ability to inhibit these pathways reinvigorates anti-tumor immune responses (60). CTLA-4 is an intracellular protein in resting T-cells. After T cell receptor engagement and a co-stimulatory signal through CD28, CTLA-4 translocates to the cell surface where it mediates inhibitory signaling to the T cell, resulting in arrest of T cell proliferation and activation (60). PD-1 is a dominant negative regulator of anti-tumor T cell effector function when it is engaged with its ligand, PD-L1 which is expressed on the surface of cancer cells. Inflammation-induced PD-L1 expression in the tumor microenvironment leads to PD-1 mediated T cell exhaustion and inhibition of anti-tumor cytotoxic T cell response (60). The first FDA approved CTLA-4 blocking antibody was ipilimumab and it showed dramatic efficacy in patients with metastatic melanoma (61). The initial FDA approved PD-1 blocking antibody was pembrolizumab which showed striking clinical efficacy in patients with advanced non-small-cell lung cancer as demonstrated in the KEYNOTE-001 trial (62). Immunotherapies with antibodies targeting immune checkpoints in these initial trials resulted in impressive results in patients with metastatic cancers.

In cervical cancer, HPV is associated with increased PD-L1 expression (63). It is estimated that over half of cervical cancers overexpress PD-L1 (64). Therefore, it was hypothesized that anti PD-1 Pembrolizumab would have a beneficial effect in patients with metastatic cervical cancer. The first phase III trial for ICIs in cervical cancer was KEYNOTE 826 (65). This was a randomized, double blind, placebo controlled study in patients with persistent, recurrent or metastatic cervical cancer. The study assessed the efficacy of pembrolizumab versus placebo in combination with standard chemotherapy plus or minus bevacizumab in a patient population that was mostly PD-L1 positive (88.6%). In the 617 patients in the intention-to-treat population, PFS was 10.4 months for the pembrolizumab group and 8.2 months for the placebo group (HR for progression 0.65, p <0.001). Overall survival at 24 months was 53% in the pembrolizumab group versus 41.7% in the placebo group (HR for death of 0.64, p<0.001). Overall the study showed that both PFS and OS significantly increased with the addition of pembrolizumab to standard chemotherapy in patients with cervical cancer (64). Based on this trial, the FDA approved pembrolizumab for PD-L1 positive recurrent or metastatic cervical cancer patients (66).

Given these improved outcomes in recurrent or metastatic cervical cancer with the use of ICIs, the phase III CALLA trial evaluated the use of the anti-PD-L1 ICI Durvalumab versus placebo with and following chemoradiotherapy (CRT) for LACC. This was a phase III, randomized, double blind, placebo controlled trial in patients with previously untreated locally advanced cervical cancer (International Federation of Gynecology and Obstetrics (FIGO) 2009 stage IB2-IIB lymph node positive or stage III/IVA regardless of nodal status). The primary endpoint was PFS. At the time of data cutoff, neither group had reached the median PFS and the study failed to meet its primary endpoint (67).

Since pembrolizumab showed activity in persistent, recurrent or advanced cervical cancer patients in KEYNOTE 826, KEYNOTE-A18 investigated the same drug with and following CRT for LACC. This study was a phase III, randomized, double blind, placebo controlled trial in patients with newly diagnosed high risk (FIGO 2014 stage IB2-IIB with node positive disease or stage III/IVA regardless of nodal status) cervical cancer. Investigators compared the addition of pembrolizumab to standard CRT followed by adjuvant pembrolizumab versus placebo with standard CRT followed by placebo maintenance. The majority of patients in this study were PD-L1 positive (95%). At 24 months, the PFS was 68% in the pembrolizumab group versus 57% in the placebo group with a HR of 0.7 (95% CI 0.55-0.89, p=0.002). OS at 24 months was 87% in the pembrolizumab group and 81% in the placebo group (HR 0.73, 95% CI 0.49-1.07) (68). PFS was significantly improved by the addition of pembrolizumab to standard of care CRT. While the difference in OS was not statistically significant in the initial analysis, follow up OS analysis demonstrated a statistically significant improvement in OS at 36 months of 82.6% in the pembrolizumab group compared to 74% in the placebo arm (p=0.004) (69). Based on the results of KEYNOTE A18, the FDA approved pembrolizumab in combination with chemoradiotherapy for the treatment of patients with FIGO 2014 stage III/IVA PD-L1 positive cervical cancer (70).

There are several ideas as to why the results of CALLA and KEYNOTE A-18 differred. First, they used a different category of ICIs (anti-PD-L1 in the CALLA trial and anti-PD-1 in the KEYNOTE A-18 trial). Cervical cancers express both PD-L1 and PD-L2 (71) so it is hypothesized that the PD-1 inhibitors, which bind to PD-1 on the T cell are able to inhibit both the PD-L1 and the PD-L2 pathways unlike the PD-L1 inhibitors which solely block the PD-L1 pathway. An exploratory subgroup analysis of CALLA showed that patients with PD-L1 tumor area positivity of 20% or more did have a reduced risk of progression in the Durvalumab group. Overall, clinical data, PD-1 inhibitors show better survival outcomes compared to PD-L1 inhibitors (72). Additionally, KEYNOTE A18 had a higher risk population with 85% being node positive and there was a requirement for a higher burden of node positivity which may have led to a better response to immunotherapy and a larger difference seen between treatment and placebo groups (68). However, given the limitations of cross trial comparisons, we are not able to definitively determine the reason for the differences in trial results.

By stimulating anti-tumor immune responses, ICIs provide a complementary approach to traditional CRT in cervical cancer (73) However, the benefits of ICIs in cervical cancer research have only proven benefit in a subset of patients as seen in KEYNOTE 826 and KEYNOTE A18 in the PD-L1 positive population (65, 68, 69). While immunotherapies have significantly improved cervical cancer patient outcomes, they can cause significant and debilitating side effects that limit their use. Systemic immune activation by ICIs put patients at risk for severe autoimmune complications that include pneumonitis, myocarditis, hepatitis, neurologic toxicities (i.e myasthenia gravis or aseptic meningitis), endocrinopathies (i.e hypo/hyperthyroidism or diabetes), pancreatitis, thyroiditis and colitis (74). Given this mechanism, immunosuppressants may be used to mitigate severe AEs (74, 75). To improve the therapeutic index of immunotherapies, more targeted immunogenic cancer cell death could lead to improved outcomes in cervical cancer that is not limited to the PD-L1 population while decreasing toxicity. Cytotoxic CRT can induce immunogenic cell death where dying cancer cells release danger-associated molecular patterns (DAMPs) signals such as ATP and HMGB1 that stimulate anti-tumor immune responses (76).

## Precision multimodal ADC-radio-immunotherapy

Concurrent chemo-radiotherapy resulted in a paradigm shifting approach to LACC over twenty years ago. Since then, radiotherapy and immunotherapy have progressed toward realizing precision oncology. IMRT/IGRT radiotherapy techniques conform IR dose delivery to MRI and PET delineated tumor targets to reduce normal tissue damage (55). While there have been significant advancements in more precise radiation delivery and the addition of ICIs to improve outcomes in LACC patients, there has not been investigation into a potentially superior, more targeted chemotherapy regimen over the past several decades. ADCs provide a solution for molecularly targeted chemo-radiotherapy with cytotoxins delivered in a spatially precise manner to cancer cells while avoiding peri-tumoral normal tissues. Cooperative tumor kill by ADC drug payloads with radiotherapy could also potentiate immunotherapies. Such multimodal ADC-radio-immunotherapy combinations may provide an opportunity to improve the therapeutic index of ADCs, radiotherapy and immune checkpoint inhibitors to not only maximize tumor control in the irradiated field but also generate systemic anti-tumor immune responses that attacks both local and metastatic disease.

Monomethyl auristatin E (MMAE) is the most common ADC payload and the drug component of Tivdak. MMAE blocks tubulin polymerization resulting in G_2_/M arrest which is the cell cycle phase most sensitive to radiotherapy (77). In cell culture radiosensitization assays, MMAE increased IR induced DNA double stranded breaks, DNA damage signaling and cell kill compared to irradiation alone (78). Thus, MMAE has the dual advantage of not only being a potent single agent cancer therapeutic but can also radiosensitize and improve irradiated tumor control. Importantly, while free MMAE radiosensitizes cells indiscriminately, antibody conjugation targets MMAE radiosensitization. Proof of concept studies were established with pre-clinical research tool compounds where MMAE was conjugated to EGFR or HER2 targeting antibodies using the cleavable MC-VC-PABC (meleimidocaproyl-valine-citrulline-p- aminobenzyloxycarbonyl) linker of all clinically approved MMAE ADCs including tisotumab-vedotin. Antibody conjugation specified MMAE radiosensitization to target-receptor enriched tumors, i.e. cetuximab conjugated MMAE radiosensitized EGFR expressing but not EGFR negative cancer cells while trastuzumab conjugated MMAE radiosensitized HER2 expressing but not HER2 negative cancer cells (77). Expanding the radiosensitizing repertoire of pre-clinical MMAE based ADCs, MMAE conjugated to anti-HER3 antibody restricted radiosensitization to HER3 expressing human cancer cells and tumor xenografts (79, 80). Progressing to FDA approved ADCs with MMAE payload, pre-clinical studies have validated therapeutic synergy of MMAE based ADCs with IR. In head and neck cancer, tisotumab vedotin added to chemo-radiotherapy improved tumor xenograft control (81). In bladder cancer, enfortumab vedotin combined with IR increased tumor xenograft control (82). Interestingly, clinical case reports of exceptional tumor responses in patients treated with radiotherapy combined with MMAE containing brentuximab-vedotin or enfortumab-vedotin have been reported (83, 84).

In completed and ongoing phase III trials of ADCs in cervical cancer, ADCs have only been studied for systemic treatment of metastatic disease (39, 44) and no large trials in cervical cancer have investigated ADCs in combination with RT or ICI therapy. Furthermore, only one early phase trial has even begun the investigation of ADCs with radiation (85). This phase II basket trial in advanced solid tumors is evaluating the RC48-ADC (disitamab vedotin), an anti-HER2 MMAE ADC in combination with hypofractionated radiation therapy. The included patients were heavily pretreated with many patients having had 10 or more prior lines of therapy. This trial also includes the use of a PD-1 or PD-L1 inhibitor ICI as a synergistic agent to promote effector T-cell activation. Patients were continued on a maintenance PD-1/L1 inhibitor after 6 cycles until disease progression or unacceptable toxicity. This study’s most recent interim results were presented as an abstract at the American Society of Clinical Oncology (ASCO) meeting in 2025. This analysis included 52 patients (10 gynecologic cancers, 10 pancreatic cancers and 32 “other” including gastric, breast and colorectal cancers). The ORR was 36.5% with 2 patients achieving a complete response that lasted nearly two years (85). These results suggest that further investigation is warranted for this type of multimodal therapy in more distinct cancer types and with alternative ADC targets. These trial results indicate that there is much room for further investigation of this treatment modality in LACC with combination therapy including RT and/or ICIs.

While concerns exist regarding the possibility of an exponential side effect profile with a multi-treatment regimen, a major benefit of multi-modal therapy is the ability to decrease drug doses to a more tolerable dose compared to the high doses required in monotherapy while maintaining similar if not improved tumor responses and quality of life (86). With an increase in use of ADCs in cancer patients, the treatment safety of ADCs in combination with radiotherapy has been evaluated in several different cancer types. A meta-analysis of the safety of trastuzumab-emtansine (a HER2 targeting ADC) in combination with radiotherapy for the loco-regional treatment of non-metastatic breast cancer showed a favorable toxicity profile (87). In metastatic breast cancer, the use of concurrent trastuzumab deruxtecan with radiotherapy was quoted to have an acceptable toxicity profile (89). A case report of patients with locally advanced bladder cancer treated with enfortumab vedotin and radiation showed no increased radiation related adverse events (88). As discussed above, there is one early phase basket trial evaluating a trimodal therapy including ADC, radiation and immunotherapy that shows promising results and concluded that the trimodal therapy had manageable safety with promising efficacy (85). While there is limited data on the safety and treatment related adverse event profile of trimodal therapy. While data is certainly limited, it appears reasonable to further assess multimodal therapy regimens in patients as an attempt to achieve improved outcomes.

Given that 30-40% of treated patients have progression or recurrence within 5 years (67), we need to find a better treatment option for these patients. We hypothesize that leveraging a new agent, such as ADCs, could both decrease the risk of recurrence and distant failure in addition to providing similar benefit for radiosensitization as proven with a systemic drug such as cisplatin. We are using a paradigm of the natural progression of drug development which has been shown to often have improved outcomes when a drug has proven benefit in the metastatic or recurrent setting and then show similar benefit in earlier stage disease like the results seen in KEYNOTE A18 where pembrolizumab was first shown to have efficacy in the metastatic or recurrent setting in KEYNOTE 826 and then was shown to improve PFS and OS when used in combination with CRT in the locally advanced setting (65, 68, 69). Therefore, it is prudent to evaluate treatment with ADCs in an earlier setting given their proven efficacy in the metastatic and recurrent setting in the phase III InnovaTV-301 trial with the ADCs tisotumab vedotin targeting tissue factor and the current phase III trial TroFuse-020 of sacitizumab tirumotecan targeting TROP-2 for recurrent or metastatic cervical cancer after promising phase II trial results with this ADC (40, 45). Perhaps using a treatment that targets micrometastatic disease as well as serving as a radiosensitizer would improve outcomes in the locally advanced setting and prevent the progression to metastatic or recurrent cervical cancer by targeting cells that are able to survive chemoradiation.

**Figure 3:**
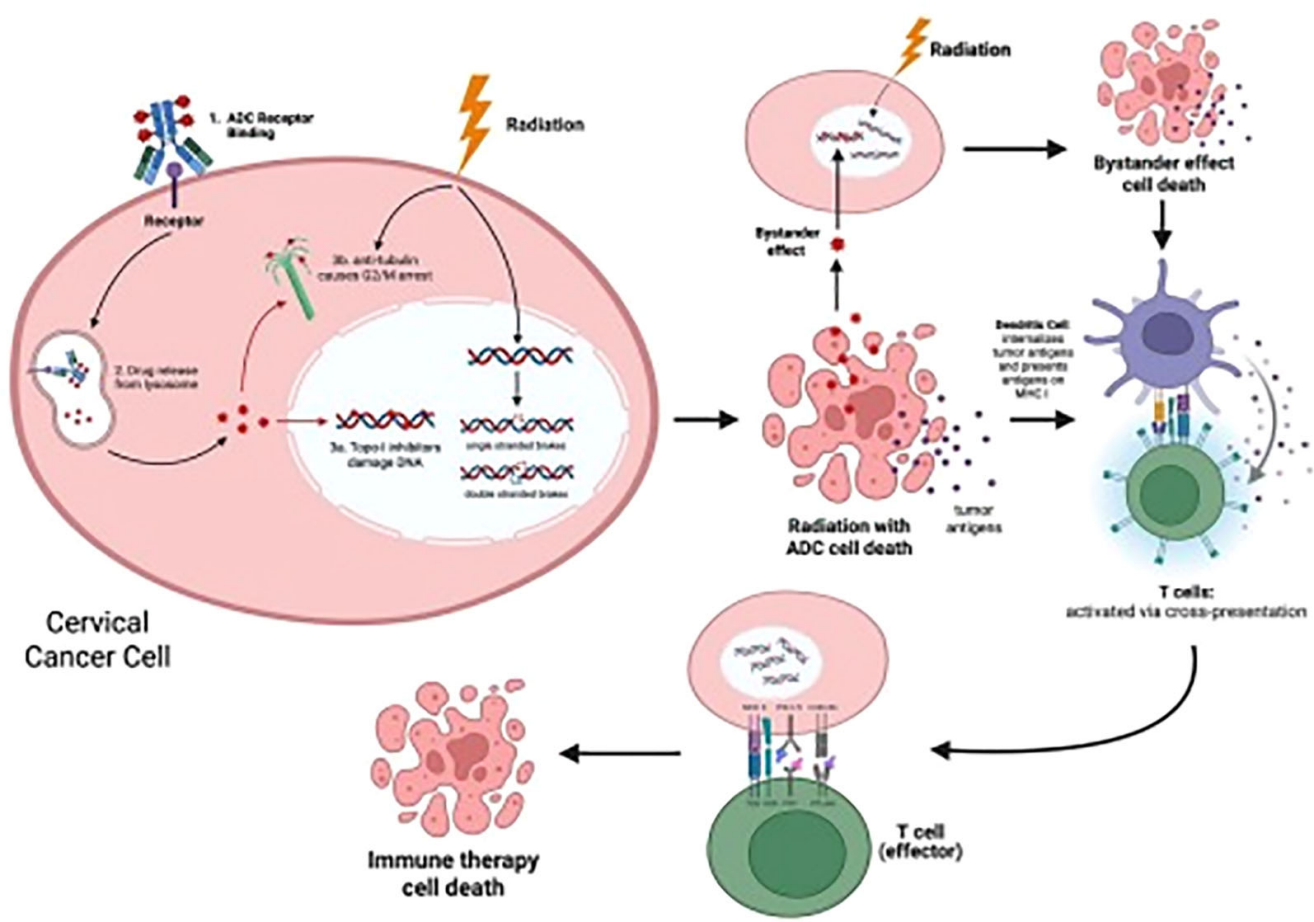
Precision multimodal ADC-radio-immunotherapy. In this proposed tri-modal cancer therapy, radiation will act by causing DNA single/double strand breaks while ADCs will bind to cancer cells expressing the ligand and be internalized to cause DNA damage via topoisomerase inhibitor or G2/M cell cycle arrest via anti-tubulin action to lead to cell death both in local tumor cells and systemic micrometastatic cells. These mechanisms of cell kill will be both additive and synergistic. ADCs will also act via bystander effect when the cancer cell dies and releases the drug payload to nearby cancer cells and target micrometastases systemically. With cell death, tumor antigens will be released and lead to a host immune response. Immune checkpoint inhibitors will then enhance the host immune response to the cancer cells to maximize cell kill and tumor control. *ADC: antibody drug conjugate.

In summary, we have seen a dramatic expansion of ADC drug development in the gynecologic cancer space, with 3 separate FDA approved therapeutic indications. Concurrently, there remains an unmet medical need in patients with locally advanced cervical cancer, advocating for a trimodal therapeutic strategy exploring ADCs as a novel radiosensitizer. Pre-clinical trials have shown radiosensitizing immunogenic cell death with release of tumor associated antigens, changing the tumor microenvironment leading to activation of the anti-tumor immune system (90). This transition would require the development of a clinical trial examining novel ADCs as a replacement of cisplatin, although platinum ineligibility in this patient population remains below 25%. Such a design would reflect a pragmatic evolution of the KEYNOTE-A18 study, with potential significant oncologic gains, as maintenance ADC and ICIs may continue to demonstrate synergistic effects (**Figure 3**).
